# Mini and Standard Percutaneous Nephrolithotomy in Obese Patients. Results from a Single-surgeon Large Series

**DOI:** 10.1016/j.euros.2024.03.011

**Published:** 2024-04-04

**Authors:** Angelo Cormio, Mario Auciello, Ugo Giovanni Falagario, Anna Ricapito, Alessandro Mangiatordi, Daniele Castellani, Andrea Benedetto Galosi, Giuseppe Carrieri, Luigi Cormio

**Affiliations:** a Urology Unit, Azienda Ospedaliero-Universitaria delle Marche, School of Urology, Faculty of Medicine, Università Politecnica delle Marche, Ancona, Italy; b Department of Urology and Organ Transplantation, University of Foggia, Foggia, Italy; c Department of Urology and Andrology, Bonomo Teaching Hospital, Andria (BAT), Italy

**Keywords:** Mini percutaneous nephrolithotomy, Standard percutaneous nephrolithotomy, Obese, Body mass index, Stone free, Blood transfusion

## Abstract

**Background and objective:**

Percutaneous nephrolithotomy (PCNL) is the recommended treatment for large or complex renal stones. This study aims to evaluate the outcomes of mini PCNL in obese and nonobese patients and to compare the outcomes of mini and standard PCNL in the obese population.

**Methods:**

We retrospectively reviewed our PCNL database to identify patients who had undergone mini (Amplatz sheath size 17.5Ch) or standard (Amplatz sheath size ≥26Ch) PCNL between 2005 and 2022. First, we compared the outcomes of the two procedures in the obese (body mass index [BMI] ≥30) and nonobese (BMI<30) patients. Second, we compared the outcomes of mini and standard PCNL in the obese population. A multivariable logistic regression analysis was performed to assess the variables associated with stone-free rate (SFR) and complications.

**Key findings and limitations:**

A total of 781 patients underwent mini PCNL; there was no difference between nonobese (578) and obese (133) patients in surgical time, number of tubeless procedures, postoperative stay, SFR, and overall complication rates. Similar outcomes were also seen in the 356 patients who had undergone standard PCNL, including 276 nonobese and 80 obese patients. The comparison of mini and standard PCNL in the obese population (213 patients) showed that mini PCNL provided significant benefits in surgical time (60 vs 94 min), SFR (85% vs 63.8%), and blood transfusion rate (2% vs 10%). The multivariable analysis confirmed that mini PCNL resulted in significantly higher odds of being stone free (odds ratio [OR] 1.79) and lower odds of having a blood transfusion (OR 0.28).

**Conclusions and clinical implications:**

Obese patients can safely undergo either mini or standard PCNL; in this series, mini performed better than standard PCNL in terms of SFR and blood transfusion rates.

**Patient summary:**

In this study, we compared the outcomes of mini and standard percutaneous nephrolithotomy (PCNL) in the obese population. We found that mini PCNL had lower surgical time and blood transfusion rate, and better stone-free rate than its standard counterpart in obese patients.

## Take Home Message

In this study, we looked at the outcomes of percutaneous nephrolithotomy (PCNL) in obese patients. We found that complications and stone-free rates (SFRs) were similar in obese and nonobese patients. Mini PCNL had a lower rate of blood transfusion and a better SFR in obese patients.

## 1 Introduction

Obesity, defined as a body mass index (BMI) of ≥30 kg/m^2^, has become a worldwide health concern for both developed and developing countries [1]. The association between obesity and kidney stone formation has a prevalence of 10-35% [2], [3].

The recommended treatment for large or complex renal or proximal ureteral stones is percutaneous nephrolithotomy (PCNL), but several factors may limit its use in obese patients. A high BMI may create difficulties with patient positioning and anesthesia, particularly in the prone position. The supine position can at least partly overcome these drawbacks. However, the prone position leads to greater kidney mobility and a further increase in stone-to-skin distance, thus requiring longer access sheaths and greater skill in creating the tract.

Studies assessing PCNL safety and efficacy in obese patients have yielded controversial results. A large single-center series assessing the impact of BMI on the outcomes of prone PCNL showed no difference in any grade of complications, length of stay, and stone-free rate (SFR) between superobese (BMI >50 kg/m^2^), normal, and overweight patients [4]. Conversely, other studies pointed out that obese patients required longer operative times and had a lower SFR [5]. Yet, obese patients were more prone to cardiovascular complications, thromboembolic events, and wound infections than overweight or normal-weight patients [6], [7]. Owing to such potential matters, urologists may be reluctant to offer standard PCNL to obese patients, particularly when stone size is between 10 and 20 mm where flexible ureteroscopy can also be performed [8], [9], [10]. In stones of these sizes, mini PCNL showed a similar SFR to standard PCNL, but with a lower complication rate [11], [12]; however, little is known about how mini PCNL performs in obese patients.

The present study aimed to compare the outcomes of mini PCNL in obese versus nonobese patients. The secondary study endpoint was comparing the outcomes of mini and standard PCNL in obese patients.

## 2 Patients methods

### 2.1 Patient population

We retrospectively reviewed our internal review board-approved database on PCNL to identify patients who had undergone mini (Amplatz sheath size 17.5Ch) or standard (Amplatz sheath size ≥26Ch) PCNL between 2005 and 2022. First, we evaluated the outcomes of the two procedures in the obese (BMI ≥30) and nonobese (BMI <30) patients. Second, we compared the outcomes of mini and standard PCNL in the obese population.

### 2.2 Surgical approach

Preoperatively, all patients underwent an abdominal computed tomography (CT) scan to assess stone volume, number, and location. Stone size was measured by gathering the longest diameter of the stone or the sum of their longest diameters in the case of multiple stones. A midstream urine culture was taken to evaluate urinary infections. According to current recommendations [13], patients with positive urine cultures received a single attempt to sterilize urine by a sensitivity-based antibiotic course; in the case of persistently positive urine cultures, they further received sensitivity-based antibiotics starting 3 d before the procedure. All patients with a negative urine culture received intravenous antibiotic prophylaxis with 2 g cefazolin and 300 mg netilmicin at anesthesia induction; thereafter, they received 1 g ceftriaxone per day intravenously until discharge provided there was an absence of infectious complications.

All procedures were carried out by one experienced surgeon (L.C.) in our supine anterolateral position [14] or the Galdakao-modified supine position.

The standard anesthesia was general until the end of 2014 and spinal since 2015. Access to the collecting system was achieved under fluoroscopic guidance using an 18G needle. The choice of the target calyx was based on stone features and the anatomy of the collecting system.

From April 2005 to September 2014, the percutaneous tract was dilated to 26-30Ch (standard PCNL) using the Amplatz Type Renal Sheath Set from Boston Scientific (Malborough, MA, USA); specifically, after having placed the 8/10 dilator sheath over the guidewire, one-step dilation was carried out to place the 26-30Ch 17-cm long Amplatz sheath. Since September 2014, we progressively shifted to mini PCNL (17.5Ch), which became routine by the end of 2015. Mini PCNL was performed using the MIP system from Karl Storz (Tuttlingen, Germany); after passing the 11Ch metallic dilator, one-step dilation was carried out to place the 17.5Ch 18-cm long “supine” Amplatz sheath. A second access was necessary in only 12 out of 1137 cases (1.1%).

Standard PCNL was performed using an 8-mm rigid nephroscope from Karl Storz, and lithotripsy was carried out with the EMS Swiss Lithoclast Master; flexible cystonephroscopes and ureteroscopes from Karl Storz with holmium laser from Lumenis were used whenever deemed necessary. Mini PCNL was performed using a 4-mm rigid nephroscope from Karl Storz, and lithotripsy was carried out using the holmium laser from Lumenis and lately the thulium fiber laser from Quanta. Independently of the laser we used, either holmium or thulium fiber laser, settings were adapted to obtain both dust (that continuously passes around the nephroscope) and 1-3 mm fragments that could easily pass through the sheath when the nephroscope is pulled back (Venturi effect).

Following stone(s) fragmentation and extraction, flexible ureteroscopy and/or flexible nephroscopy was carried out in all cases to check for stone clearance.

The preferred exit strategy was a tubeless procedure, thus avoiding a nephrostomy tube but placing an indwelling single- or double-J ureteral catheter. In the case of standard PCNL, we usually applied Tachosil to better seal the tract [15]. Nephrostomy tubes were, however, used whenever deemed necessary, mainly in case of relevant bleeding or a risk of sepsis.

All patients underwent KUB x-ray and renal ultrasonography at 1 and 3 mo postoperatively to assess residual fragments. A CT scan was used whenever the presence of residual fragments was suspected. Patients with residual fragments ≤4 mm were considered stone free. Perioperative complications were assessed using the Clavien classification system adjusted for PCNL [16]. Infectious complications were defined as systemic inflammatory response syndrome or fever >38°C lasting >24 h, and/or positive urine or blood culture.

### 2.3 Statistical analysis

Continuous variables are reported as medians and interquartile ranges and tested by the Kruskal-Wallis test, while categorical variables are reported as absolute numbers and percentages and tested by the chi-square test. A multivariable logistic regression analysis was performed to assess the variables associated with SFR and major complications (ie, Clavien grade >2). Data are presented as odds ratio (OR) and 95% confidence interval (CI). All statistical tests were performed using Stata 16.1 (StataCorp LP, College Station, TX, USA). All tests were two sided, with a significance level set at *p* < 0.05.

## 3 Results

Among the 781 patients who underwent mini PCNL, 578 were nonobese, whereas 133 were obese. Table 1 summarizes the preoperative characteristics, surgical data, and outcomes. Although obese patients were more likely to have diabetes mellitus and had significantly greater American Society of Anesthesiologists (ASA) scores and stone sizes, there was no difference in the remaining preoperative variables. Interestingly, there was also no significant difference between the two groups in surgical time, number of tubeless procedures, postoperative stay, SFR, and complications rate.

**Table 1:** Preoperative characteristics, surgical data, and outcomes of patients who had mini PCNL

	BMI <30 (*N* = 578)	BMI ≥30 (*N* = 133)	*p* value
Age (yr), median (IQR)	54.7 (45.5, 65.4)	56.8 (49.5, 64.0)	0.2
Female gender, *n* (%)	279 (48.3)	75 (56.4)	0.091
BMI, median (IQR)	25.9 (24.0, 27.3)	32.0 (31.0, 36.0)	**<0.0001**
Diabetes mellitus, *n* (%)	76 (13.1)	31 (23.3)	**0.003**
Chronic bacteriuria, *n* (%)	77 (13.3)	18 (13.5)	0.9
ASA, *n* (%)			
1-2	527 (91.2)	101 (75.9)	**<0.0001**
3-4	51 (8.8)	32 (24.1)	
Cumulative stone size (mm), median (IQR)	21.0 (17.0, 28.0)	25.0 (20.0, 30.5)	**0.003**
Stone features, *n* (%)			
Single	249 (43.1)	52 (39.1)	0.7
Multiple	201 (34.8)	51 (38.3)	
Staghorn	128 (22.1)	30 (22.6)	
Surgical time (min), median (IQR)	60.0 (50.0, 80.0)	60.0 (50.0, 75.0)	1
Tubeless, *n* (%)	498 (86.3)	110 (83.3)	0.4
Stone free, *n* (%)	457 (79.1)	113 (85.0)	0.12
Clavien, *n* (%)			
0	379 (65.6)	92 (69.2)	0.4
1-2	141 (24.4)	27 (20.3)	0.3
≥3	58 (10.0)	14 (10.5)	0.9
24 h HB loss (g/dl), median (IQR)	1.2 (0.3, 2.0)	1.2 (0.1, 2.1)	0.8
Blood transfusion, *n* (%)	13 (2)	3 (2)	1
Infectious complications, *n* (%)	67 (11.6)	14 (10.5)	0.7
Postop. hospital stay (d), median (IQR)	3.0 (2.0, 4.0)	3.0 (2.0, 4.0)	0.6

ASA = American Society of Anesthesiologists; BMI = body mass index; HB = hemoglobin; IQR = interquartile range; PCNL = percutaneous nephrolithotomy.

Table 2 summarizes the preoperative characteristics, surgical data, and outcomes of patients who had undergone standard PCNL. There were 276 patients in the obese group and 80 in the normal-weight group. Obese patients had significantly greater ASA scores and stone sizes than nonobese ones, but again there was no significant difference in intra- and postoperative outcomes.

**Table 2:** Preoperative characteristics, surgical data, and outcomes of patients who had standard PCNL

	BMI <30 (*N* = 276)	BMI ≥30 (*N* = 80)	*p* value
Age (yr), median (IQR)	54.7 (41.6, 62.9)	51.2 (44.1, 61.4)	0.6
Female gender, *n* (%)	131 (48.3)	56 (70.0)	**0.001**
BMI, median (IQR)	26.0 (24.5, 27.1)	33.0 (31.0, 36.0)	**<0.0001**
Diabetes mellitus, *n* (%)	19 (7.1)	10 (12.5)	0.13
Chronic bacteriuria, *n* (%)	24 (9.0)	6 (7.5)	0.7
ASA, *n* (%)			
1-2	241 (90.3)	62 (77.5)	**0.003**
3-4	26 (9.7)	18 (22.5)	
Cumulative stone size (mm), median (IQR)	24.0 (19.0, 30.0)	26.0 (20.0, 30.5)	**0.041**
Stone features, *n* (%)			
Single	125 (46.8)	29 (36.2)	0.2
Multiple	95 (35.6)	32 (40.0)	
Staghorn	47 (17.6)	19 (23.8)	
Surgical time (min), median (IQR)	90.0 (60.0, 120.0)	94.0 (62.5, 120.0)	0.4
Tubeless, *n* (%)	201 (75.3)	64 (80.0)	0.4
Stone free, *n* (%)	190 (71.2)	51 (63.8)	0.2
Clavien, *n* (%)			
0	167 (62.5)	50 (62.5)	1
1-2	83 (31.1)	24 (30.0)	0.9
≥3	17 (6.4)	6 (7.5)	0.7
24 h HB loss (g/dl), median (IQR)	1.1 (0.0, 2.7)	1.0 (0.0, 2.2)	0.8
Blood transfusion, *n* (%)	18 (7)	8 (10)	0.3
Infectious complications, *n* (%)	25 (9.4)	6 (7.5)	0.6
Postop. hospital stay (d), median (IQR)	3.0 (2.0, 4.0)	3.0 (2.0, 4.0)	0.9

ASA = American Society of Anesthesiologists; BMI = body mass index; HB = hemoglobin; IQR = interquartile range; PCNL = percutaneous nephrolithotomy.

Table 3 shows the characteristics and outcomes of obese patients who had either standard or mini PCNL. Despite being older, mini PCNL patients had significantly shorter surgical time (60 vs 94 min, *p* < 0.0001), higher SFR (85% vs 63.8%, *p* = 0.0004), and lower blood transfusion rate (2% vs 10%, *p* = 0.013).

**Table 3:** Preoperative characteristics, surgical data, and outcomes of mini and standard PCNL in obese patients

	Standard PCNL (*N* = 80)	Mini PCNL (*N* = 133)	*p* value
Age (yr), median (IQR)	51.2 (44.1, 61.4)	56.8 (49.5, 64.0)	**0.014**
Female gender, *n* (%)	56 (70.0)	75 (56.4)	**0.048**
BMI, median (IQR)	33.0 (31.0, 36.0)	32.0 (31.0, 36.0)	0.6
Diabetes mellitus, *n* (%)	10 (12.5)	31 (23.3)	0.053
Chronic bacteriuria, *n* (%)	6 (7.5)	18 (13.5)	0.2
ASA, *n* (%)			
1-2	62 (77.5)	101 (75.9)	0.8
3-4	18 (22.5)	32 (24.1)	
Cumulative stone size (mm), median (IQR)	26.0 (20.0, 30.5)	25.0 (20.0, 30.5)	0.5
Stone features, *n* (%)			
Single	29 (36.2)	52 (39.1)	0.9
Multiple	32 (40.0)	51 (38.3)	
Staghorn	19 (23.8)	30 (22.6)	
Surgical time (min), median (IQR)	94.0 (62.5, 120.0)	60.0 (50.0, 75.0)	**<0.0001**
Tubeless, *n* (%)	64 (80.0)	110 (83.3)	0.5
Stone free, *n* (%)	51 (63.8)	113 (85.0)	**0.0004**
Clavien, *n* (%)			
0	50 (62.5)	92 (69.2)	0.2
1-2	24 (30.0)	27 (20.3)	
≥3	6 (7.5)	14 (10.5)	
24 h HB loss (g/dl), median (IQR)	1.0 (0.0, 2.2)	1.2 (0.1, 2.1)	1
Blood transfusion, *n* (%)	8 (10)	3 (2)	**0.013**
Infectious complications, *n* (%)	6 (7.5)	14 (10.5)	0.5
Postop. hospital stay (d), median (IQR)	3.0 (2.0, 4.0)	3.0 (2.0, 4.0)	0.6

ASA = American Society of Anesthesiologists; BMI = body mass index; HB = hemoglobin; IQR = interquartile range; PCNL = percutaneous nephrolithotomy.

A multivariable analysis (Table 4) showed that BMI was not associated with higher odds of complications. Mini PCNL was significantly associated with higher odds of being stone free (OR 1.79, 95% CI 1.33-2.42, *p* < 0.001) and lower odds of having blood transfusion (OR 0.28, 95% CI 0.15-0.53, *p* < 0.001). Chronic bacteriuria and staghorn stones were significantly associated with higher odds of both infectious and major complications.

**Table 4:** Multivariable analysis of factors associated with stone-free status, blood transfusion, and Clavien >2 and infectious complications

Covariate	Stone-free status			Blood transfusion			Clavien >2 complications			Infectious complications		
	OR	95% CI	*p* > |*z*|	OR	95% CI	*p* > |*z*|	OR	95% CI	*p* > |*z*|	OR	95% CI	*p* > |*z*|
Age (yr)												
Per unit	1.00	0.99, 1.01	0.945	1.00	0.98, 1.02	0.787	1.00	0.99, 1.02	0.627	1.00	0.98, 1.01	0.721
Gender, *n* (%)												
Male	1			1			1			1		
Female	0.91	0.67, 1.22	0.512	1.48	0.77, 2.84	0.234	1.26	0.80, 1.98	0.317	1.06	0.70, 1.60	0.790
BMI												
Per unit	1.00	0.97, 1.03	0.863	1.01	0.95, 1.08	0.708	0.98	0.93, 1.02	0.293	0.98	0.94, 1.02	0.336
Diabetes												
No	1			1			1			1		
Yes	1.28	0.80, 2.05	0.307	2.17	0.97, 4.87	0.060	1.41	0.78, 2.56	0.250	1.41	0.81, 2.47	0.225
Stone features, *n*												
Single	1			1			1			1		
Multiple	0.91	0.66, 1.27	0.578	1.02	0.48, 2.14	0.962	1.58	0.93, 2.69	0.088	1.70	1.05, 2.76	**0.031**
Staghorn	0.83	0.57, 1.21	0.328	1.68	0.77, 3.69	0.193	2.37	1.37, 4.11	**0.002**	2.22	1.32, 3.72	**0.003**
Chronic bacteriuria												
No	1			1			1			1		
Yes	0.69	0.45, 1.07	0.099	0.17	0.02, 1.26	0.082	2.76	1.63, 4.67	**<0.001**	2.91	1.77, 4.80	**<0.001**
ASA score												
1-2	1			1			1			1		
3-4	1.27	0.78, 2.05	0.338	1.12	0.43, 2.87	0.821	1.39	0.75, 2.58	0.298	1.18	0.65, 2.16	0.581
Amplatz												
Standard	1			1			1			1		
Mini	1.79	1.33, 2.42	**<0.001**	0.28	0.15, 0.53	**<0.001**	1.39	0.84, 2.30	0.195	1.16	0.74, 1.82	0.514

ASA = American Society of Anesthesiologists; BMI = body mass index; CI = confidence interval; OR = odds ratio.

## 4 Discussion

The present study demonstrated that mini PCNL had similar outcomes in obese and nonobese patients. Specifically, there was no difference in SFR, although obese patients had a significantly larger stone size, and there was no difference in the rate of complications, although the proportion patients with of ASA 3 and 4 scores was significantly higher in the obese population.

To our knowledge, only one study has addressed the outcomes of mini PCNL in obese patients previously [17]. This study included 67 patients who underwent mini PCNL (access sheath ≤22Ch); 34 were classified as nonobese (BMI <30) and 33 as obese (BMI ≥30). In line with our findings, there was no statistically significant difference in all tested outcomes, including SFR and complication rate. Differently from our study, the nonobese patients had a significantly higher overall stone burden (median stone size 18 vs 15 mm, *p* = 0.02). The authors pointed out that such findings were consistent with those reported in a series of 1152 standard PCNL cases treated at their institution with no difference in surgical time, SFR, or secondary procedures among obese and even superobese patients (BMI >50). Again, these findings are in line with the results of those patients who had standard PCNL in our series.

Previous large multicenter studies focusing on PCNL efficacy and safety in obese patients yielded somehow different results. A retrospective analysis of 90 529 patients who had PCNL between 1998 and 2010 [18] identified 9300 obese patients and compared their outcomes with nonobese patients. Overall, there was no significant difference between obese and nonobese patients in terms of overall complications (21.6% vs 22.0%, *p* = 0.3) and transfusion rates (4.3% vs 4.0%, *p* = 0.1), but the obese group had a significantly higher rate of sepsis (1.7% vs 1.3%, *p* = 0.009) as well as of respiratory (3.0% vs 2.5%, *p* = 0.002) and vascular (0.3% vs 0.2%, *p* = 0.007) complications. The largest multicenter prospective study gathering data from 5803 patients treated at 96 centers worldwide [5] showed that PCNL was safe in obese patients, since there was no difference in the length of stay, blood transfusion, and overall complication rates even if obese patients had a significantly longer surgical time and a lower SFR. It is worth mentioning that both studies suffer unavoidable biases in caseload, surgeon experience, and surgical technique. Moreover, the vast majority of patients had undergone standard PCNL up to 2010, but the aforementioned studies do not provide information on developments that occurred in standard and particularly mini PCNL over the last decade.

The novelty of our study is the comparison of standard and mini PCNL outcomes in obese patients. We found that mini PCNL was associated with a shorter surgical time yet a greater SFR. There was no difference in the rates of uneventful procedures (Clavien 0), as well as of minor (Clavien <2) and major (Clavien >2) complications. That said, mini PCNL was associated with a significantly lower rate of blood transfusion (Clavien 2), which is one of the expected benefits of sheath miniaturization. A recent randomized controlled trial [19] comparing the outcomes of standard (30Ch) and mini (16.5Ch) PCNL reported similar surgical time and SFR, but a significantly lower rate of blood transfusion for mini PCNL (1.2% vs 9.8%, *p* = 0.03). Another large randomized controlled trial comparing standard (24Ch) and mini (18Ch) PCNL in the setting of 20-40-mm renal stones [20] showed the same SFR (86%), and similar surgical time (36 vs 35 min) and blood transfusion rate (1.1% vs 1.3%) for the two procedures, but significantly lower hemoglobin drop and postoperative pain for mini PCNL.

Our multivariable analysis showed that obesity was not associated with a greater risk of blood transfusion or major (Clavien >2) complications. Yet, mini PCNL was associated with higher odds of being stone free and lower odds of having blood transfusions. Therefore, our study clearly points out that obese patients can safely undergo PCNL and that they can benefit even more from mini PCNL in terms of a better SFR and lower blood transfusion rate.

It should, however, be acknowledged that BMI may not be the most relevant anthropometric parameter of obesity for PCNL. A recent single-center study [21] including 150 patients tested the impact of visceral fat and abdominal circumference on PCNL outcome. SFR was not affected by visceral fat area or BMI, but only by abdominal circumference. Unfortunately, the study did not address the issue of PCNL safety, nor did it classify patients according to their BMI.

A strong point of our study is the large and well-distributed population. Together with the inclusion of consecutive patients, it provides a reliable picture of real-life clinical practice of a referral center, including the effects of shifting from standard to mini PCNL for all cases. That said, the present study also has some limitations. First, we did not record further anthropometric parameters such as fat mass index, visceral fat area, abdominal circumference, and skin-to-stone distance, which could all affect the outcomes of both standard and mini PCNL. Indeed, the assessment of obesity anthropometric parameters that can be more relevant for PCNL would undoubtedly deserve further investigation. Second, the comparison of standard and mini PCNL in the obese population might be affected by the time frame; indeed, mini PCNL was carried out from 2014 onward, when the progressive increase in surgeon’s experience might have led to better results in terms of SFR. Finally, SFR evaluation could have been more robust if a CT scan was used in all cases, but given that kidney stone disease is often a chronic condition with frequent recurrence, relying solely on CT scans would substantially increase radiation exposure for affected patients, particularly in obese patients who are at a higher risk of recurrence [22]. Moreover, the inherent radiation exposure of CT is a further concern, particularly considering that <8% of patients undergoing CT for urolithiasis were imaged using a low-dose protocol [23].

## 5 Conclusions

The present study provides clear evidence that obese patients can safely undergo PCNL and that they can benefit mostly from mini PCNL in terms of both a better SFR and a lower blood transfusion rate. Future prospective studies, possibly multicentered and incorporating a wider range of anthropometric parameters, would be beneficial to corroborate these findings and enhance their applicability in diverse clinical settings.

  ***Author contributions*:** Angelo Cormio had full access to all the data in the study and takes responsibility for the integrity of the data and the accuracy of the data analysis.

  *Study concept and design*: L. Cormio.

*Acquisition of data*: Auciello, Ricapito, Mangiatordi.

*Analysis and interpretation of data*: L. Cormio, A. Cormio, Castellani.

*Drafting of the manuscript*: A. Cormio, Castellani, L. Cormio.

*Critical revision of the manuscript for important intellectual content*: Galosi, Carrieri, L. Cormio.

*Statistical analysis*: Falagario.

*Obtaining funding*: None.

*Administrative, technical, or material support*: None.

*Supervision*: L. Cormio.

*Other*: None.

  ***Financial disclosures***: Daniele Castellani certifies that all conflicts of interest, including specific financial interests and relationships and affiliations relevant to the subject matter or materials discussed in the manuscript (eg, employment/affiliation, grants or funding, consultancies, honoraria, stock ownership or options, expert testimony, royalties, or patents filed, received, or pending), are the following: None.

  ***Funding/Support and role of the sponsor*:** None.
